# The perceptions of food service staff in a nursing home on an upcoming transition towards a healthy and sustainable food environment: a qualitative study

**DOI:** 10.1186/s12877-023-04493-x

**Published:** 2023-11-28

**Authors:** Femke A. Hoefnagels, Olga N. Patijn, Marieke J. G. Meeusen, Marieke C. E. Battjes-Fries

**Affiliations:** 1 Department of Health and Nutrition, Louis Bolk Institute, Kosterijland 3-5, Bunnik, 3981 AJ the Netherlands; 2 Department IxD, University of Twente, Drienerlolaan 5, Enschede, 7522 NB The Netherlands; 3 Wageningen Economic Research, Wageningen University & Research, P.O. Box 29703, The Hague, 2502 LS The Netherlands

**Keywords:** Transition, Sustainable, Healthy, Food Environment, Nursing home, Qualitative research, Barriers, Facilitators

## Abstract

**Background:**

Healthy and sustainable food environments are urgently needed, also in nursing and residential care homes. Malnutrition in care homes is becoming an increasing problem as populations worldwide are ageing and many older people do not consume sufficient protein, fibre, fruit, and vegetables. Nursing homes also often experience a lot of food waste. A transition in the food environment like a nursing home, involves the participation of facility management and food service staff members. This study aims to map out their perceived barriers and facilitators for this transition.

**Methods:**

A qualitative study using semi-structured interviews was conducted with food service staff members (n = 16), comprising of kitchen staff (n = 4), wait staff (n = 10), and facility management (n = 2) of two nursing homes in the Netherlands. Thematic analysis was used to derive content and meaning from transcribed interviews.

**Results:**

Four main themes were identified. Theme 1: ‘Communication, transparency and accountability in the chain’, highlighting the lack of effective communication flows and a fragmented overview of the food service chain as a whole. Theme 2: ‘Understanding, knowledge and ability of the concepts healthy and sustainable’, revealing the gap in staff’s understanding of these abstract concepts, despite perceiving themselves as having sufficient knowledge and ability. Theme 3: ‘The pampering service mind-set’, highlighting the contradiction in the staff’s shared goal of proving the highest quality of life for residents while also pampering them in ways that may not align with promoting healthy and sustainable food choices. Theme 4: ‘Transition is important but hard to realize’, describing the barriers such as existing routines and a lack of resources as challenges to implementing changes in the food service.

**Conclusions:**

Facilitators to transitioning nursing homes towards a healthy and sustainable food environment as perceived by staff members included transparent communication, accountability in the food supply chain, staff’s perceived ability and shared goal, while barriers included lack of understanding of the concepts healthy and sustainable, the current pampering mindset, and top-down decision-making. These findings provide valuable insights for nursing homes seeking to transition towards a healthier and more sustainable food environment.

**Supplementary Information:**

The online version contains supplementary material available at 10.1186/s12877-023-04493-x.

## Background

Malnutrition is a highly prevalent problem among nursing and residential homes and is becoming an increasing problem as populations worldwide are ageing and reside more frequently in nursing and residential care homes [1, 2]. In the Netherlands, where over 115 thousand older people reside in long-term care, the malnutrition prevalence rate is around 16% and the risk of developing malnutrition is 50% [3, 4]. Similar healthcare challenges can be found in the rest of the world, with a malnutrition prevalence of 17.5% in nursing homes and a risk of malnutrition of 48.0% [5]. Older people often deal with the loss of taste, decreased appetite, pain and/or nausea, or swallowing and chewing problems [6, 7]. Many older people do not consume sufficient protein, fibre, fruit, and vegetables despite living in a nursing home and getting all their meals catered [8–10]. Nutrition does not always have a structural position in the care policy of the nursing home [10]. Malnutrition in nursing homes needs to be addressed because it has a direct, negative, influence on the quality of life of residents [11]. When a person is malnourished, he or she is more prone to infections, inflammation, weakened muscles, and fatigue [1]. Malnutrition can lead to loss of muscle mass, delayed wound healing, decrease in resilience, decreased muscle strength, increased falling risk, and increased postoperative complications [12–14].

In addition to malnutrition, nursing homes also often experience a lot of food waste. Approximately 30% of the food that is suitable for consumption is thrown away in nursing homes [15]. In hospitals, food can make up to 50% of the total waste generated [16]. Possible causes for food waste in this sector are the nature of the patient population and the foodservice systems [17]. Malnutrition-related complications, such as reduced energy and protein intake despite the quantity offered is in accordance with the food guidelines, are related to high food wastage [18]. Therefore, it is evident that addressing both malnutrition and sustainable food practices within nursing and residential care homes is essential; not only to enhance the health of residents but also to minimize unnecessary waste and promote a more environmentally conscious approach to food service.

The causes of malnutrition and food waste in nursing homes are linked to the social and physical food environment. Bianchi and colleagues state that dietary change interventions, for example to stimulate healthier eating behaviour or sustainable behaviour, can have the greatest impact when they are conducted in ‘closed settings’ like nursing homes [19]. Characteristics of a closed setting includes: the food supply is determined by a supplier, caterer, and/or another party; set mealtime hours and limited menu options and fewer external influences [20]. Due to the limited possibilities for residents of nursing homes to buy and consume food from elsewhere than within the food environment that is provided by the nursing home, it can be relatively easy to guide these residents towards making healthier choices [21]. A review by Taufik, Jaspers, Bouwman, and Reinders [20] revealed that the main barriers surrounding nutrition interventions in closed settings were lack of time, lack of staff capacity, and inflexibility of the food service system. The main supporting factors were the satisfaction of the staff with the intervention and the involvement with patients as a result of the intervention [20]. A transition involves the participation of facility management and food service staff members. Previous studies showed that the effectiveness of interventions is, at least partly, dependent on the extent to which they are accepted and implemented by these staff members [22].

There is a need to provide insight into the role of employees within the transition by enhancing the depth of understanding of staff perceptions and their knowledge on the topic. A more in depth understanding regarding the perceptions and knowledge of food service staff is needed since they are the ones navigating the transition toward fostering a healthier and more sustainable food environment. Qualitative research may be useful in illuminating gaps in understanding, where interviewees do not raise important issues or practices unless they are prompted [23]. Therefore, this study aims to map out the perceived barriers and facilitators by food service staff in nursing homes to establish a transition towards a healthier and sustainable food environment.

## Methods

### Study design

Semi-structured interviews were conducted to explore facilitators and barriers to the transition towards a healthier and sustainable food environment. The guidelines of the Netherlands Nutrition Centre were held as the definition for a healthy food environment [24]. Sustainable was defined as fresh, unprocessed, locally produced food and reducing food waste and disposables. A topic guide was used for the semi-structured interviews (appendix 1). The topic guide included the following domains: *the current food situation, knowledge about nutritional needs for older people, sustainable food, food waste, and motivation for a transition*.

**Fig. 1:**
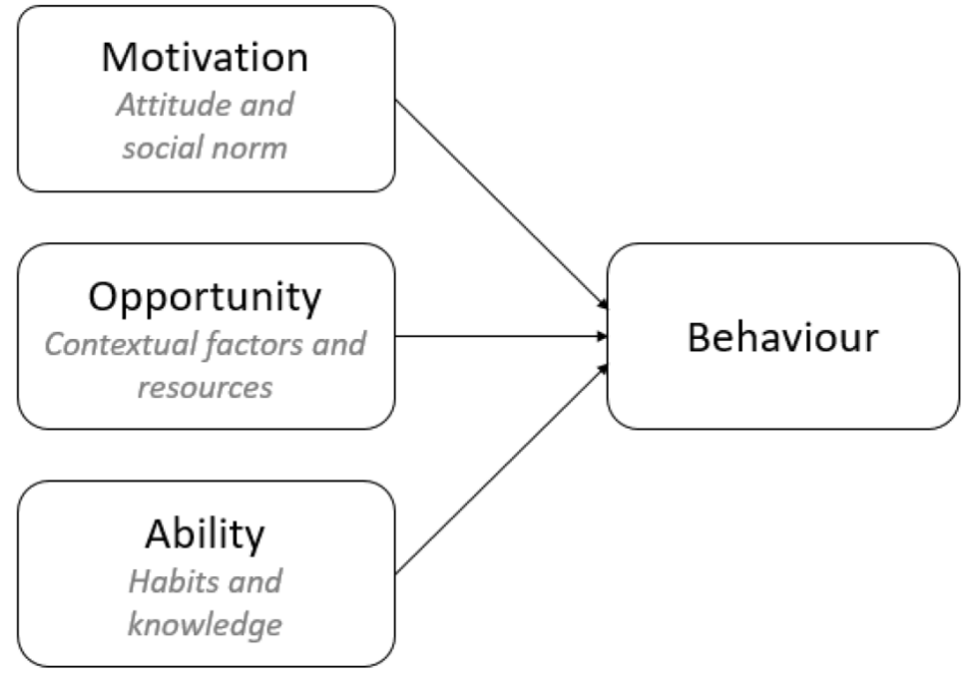
MOA framework [25]

The Motivation-Opportunity-Ability (MOA) framework functioned as the underlying structure for preparation of the interview guide and analysing the data, see Fig. 1 [25]. This framework comprises three interconnected factors, each playing a vital role in understanding and influencing behaviour. The first factor is motivation, which can be defined as goal-directed arousal. Motivation is divided into two determinants; attitude, in other words personal motivations, and the social norm, what everyone thinks others expect from them [26]. Opportunity is defined as the extent to which individuals can process information without any environmental restrictions, the contextual factors and products available to perform the behaviour [27]. Lastly, ability is defined as the capabilities available to individuals to behave in a certain manner [28]. Ability is driven by existing habits and available knowledge. Ability relates to internal skills, whereas opportunity relates to external environmental restrictions [26]. These three interwoven factors, motivation, opportunity, and ability, collectively shape and influence how individuals behave [29]. By employing the MOA framework in our research, we gain a comprehensive understanding of how these factors interact to impact the behaviour of food service staff in nursing homes, particularly regarding the transition to a healthier and more sustainable food environment. Hence, various studies have classified drivers of and barriers to sustainable consumer behaviour and health behaviours according to the MOA-framework [30–34].

### Study population

The current study is carried out at two nursing home locations from the same care group, situated in Zaandam, the Netherlands, employing in total more than 600 staff members. One centralized kitchen located indoors at location A provides over 450 warm meals per day for the residents of both locations. Meals are either consumed in the restaurant of both locations or in the residents’ own room. The residents are older people who have medical indication that they need support with their ADL (Activities of Daily Living) for somatic or psychogeriatric reasons. The majority of the residents receive all their meals via the nursing home.

The nursing homes’ management stated the ambition to create a healthier and more sustainable food environment. This research was done to map the barriers and facilitators prior to starting this transition. The food service staff consists of facility management, kitchen staff, wait staff and welfare staff involved in the food supply. Purposive sampling was used and recruitment was not based on data saturation. All types of food service staff were invited to participate and offered the opportunity to ask questions beforehand. There was no pre-established relationship between the researchers and the staff, except that the staff had seen the researchers in the nursing homes and observing the dining room. During the researchers’ presence for two months, the staff was aware of the research, its background, and the researchers’ independent position. If the staff agreed to take part, a mutually convenient time was arranged for an interview. It was made clear that participation was entirely voluntary, and they could withdraw at any time. All participating staff provided informed consent. This study was exempted from METC approval by the medical ethical review committee METC-WU.

### Data collection

Each semi-structured interview lasted approximately 15–35 min and the interviews were held in the residential care home where the participating employee worked to facilitate participants’ schedules. The employees were interviewed face-to-face, with no one else present, or by telephone if a face-to-face meeting was not possible. Most interviews were audio-recorded and transcribed verbatim, with participant information anonymized at transcription. From three interviewees we only had short reports on the interviews because they did not give consent to audiotape the interview. Interviews were conducted in Dutch and quotations were translated into English. Brief field notes were taken by the interviewer and used alongside the interview transcript during the analysis phase to provide additional context. The interviews were conducted between April and June 2021.

### Data analysis

The interviews were transcribed and anonymised before being uploaded to Atlas.ti 9.0, qualitative analyses software. A six-phase analysis process as per Braun and Clarke was followed, including familiarization with and immersion in the data, coding, generating initial themes, reviewing themes, defining and naming themes, and writing up [35]. All transcripts were initially coded by one author (FH), who summarized sentences with a word or words that encapsulated the meaning. All these codes were subsequently reviewed and discussed with another author (OP) to reach a consensus on coding decisions and the assignment of codes to sub-themes. Factors that were considered to be facilitators if their presence promoted the implementation of or adherence to the transition. Factors were considered as barriers if they impede implementation of or adherence to the transition. The same factor could be both a barrier and a facilitator. If the presence of a factor was a facilitator, its absence was considered a barrier.

Within this iterative process, a broader overarching theme was identified, and links between these and the subthemes were identified and deliberated upon by both authors. After the analysis process, the participants were given the opportunity to access and review the full transcript of their coded interviews, along with a summary of the emergent themes to determine whether the codes and themes were true to their experience, although none of them opted to take advantage of this option. Illustrative quotes representing themes and sub-themes were extracted from the transcription and a narrative description of findings was presented.

## Findings

### Participant characteristics

Sixteen employees from four different food disciplines were interviewed. Facility management is responsible for the overall administration and operation of the entire nursing home. The kitchen staff work in the kitchen which is located in location A. Their responsibilities include menu planning, meal preparation, and ensuring that meals align with dietary guidelines and safety standards, but they have no direct contact with the residents. Wait staff at location A is responsible for serving meals to residents and providing assistance during meal times. Their tasks encompass setting up the dining area, taking the order, serving food, assisting residents with their meals, and maintaining a pleasant dining atmosphere. Wait staff at location B is similar to their counterparts at location A, whereby the welfare staff members at location B are only responsible for a few residents rather than the entire dining area. The male/female distribution was almost even. The employees’ experience working in residential care ranged between 1 and 26 years (Table 1).

**Table 1:** Participant characteristics of the food service staff (n = 16)

Characteristics	N (%)
Sex	
*Men* *Women*	9 (56.3%) 7 (43.8%)
Years working in residential care (median, range)^A^	8 (1–26)
Practice type	
*Facility management* *Kitchen staff location A* *Wait staff location A* *Wait staff location B* ^*B*^	2 (12.5%) 4 (25.0%) 3 (18.8%) 7 (43.8%)

^*A*^ *Years working of three employees are missing.*

^*B*^ *Including two welfare staff members.*

### Themes

Four main themes emerged from the data (Table 2): (1) Communication, transparency, and accountability in the chain; (2) Understanding, knowledge and ability of the concepts healthy and sustainable; (3) A pampering service mind-set; and (4) Transition is important but hard to realize.

**Table 2:** Summary of main themes

*Theme*	*Details*
1. Communication, transparency, and accountability in the chain	This theme delves into the intricacies of communication within the organization, revealing a primarily one-way flow of information, a noticeable absence of a feedback loop, and a lack of transparency and accountability between the staff members. It highlights the need to establish robust channels for information exchange and the importance of a more responsive and transparent communication structure.
*2.* Understanding, knowledge and ability of the concepts healthy and sustainable	Within this theme, the multifaceted understanding of the concepts healthy and sustainable held by the staff members are explored. We delve into their actual knowledge on these concepts and their perceived ability to contribute in the transition towards a more healthy and sustainable food environment. This theme uncovers the complex interplay of ideas and perceptions surrounding health and sustainability within the organization.
3. A pampering service mind-set	This theme covers the interplay between shared common goal of staff to provide the highest quality of life for the residents. However, it also explains the challenge of respecting residents’ experienced freedom of choice within a residential setting, revealing a delicate balance between ‘providing support’ and autonomy.
4. Transition is important but hard to realize	This theme underscores the intricate the journey towards change and improvement, highlighting the obstacles that stand in the way. It uncovers the influence of established routines and emphasizes external and practical factors as significant barriers to achieving a successful transition. The theme vividly illustrates the obstacles encountered on the path to achieving a transition.

### Theme 1: communication, transparency and accountability in the chain

**Fig. 2:**
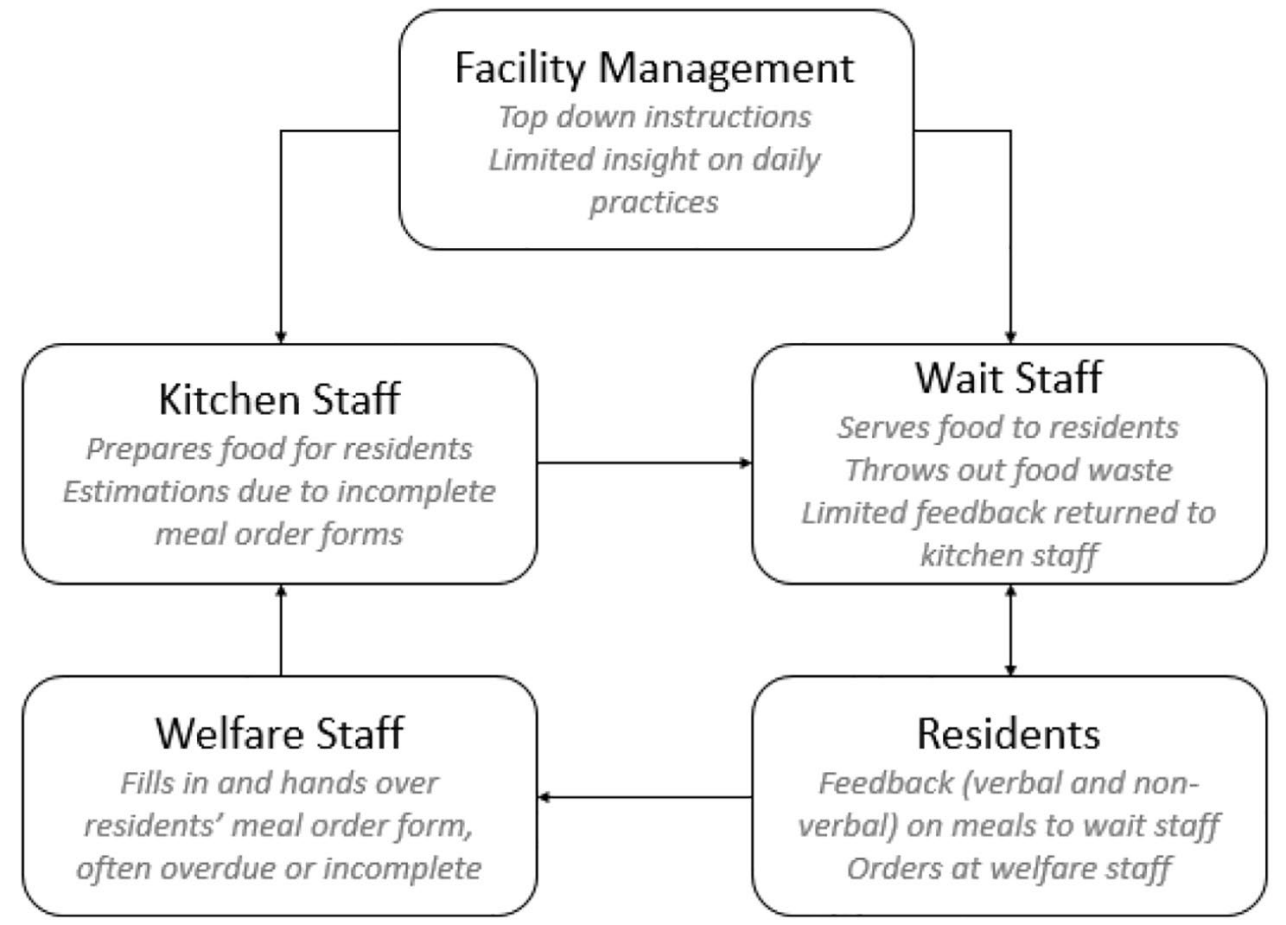
Food-related communication flow in the nursing homes

The food service staff in the studied nursing homes perceive communication to be primarily one-way, with a lack of feedback loop present. These findings are illustrated in Fig. 2.

The broken feedback loop is apparent in the way that facility management makes top-down policy decisions without direct input from kitchen and wait staff and it is they who have to implement it. Kitchen staff members indicated that they feel not involved in the choices that are made. This lack of involvement in the decision-making process is further perpetuated by the food supply chain process, in which kitchen staff do not have direct interaction with residents and only one-way communication with wait staff. Feedback on food preferences, amount served, and quality is not incorporated into the whole supply chain process. Additionally, the communication between staff members responsible for ordering food and those responsible for preparing it is inadequate, with order forms being handed over too late or incomplete. This leads to gross overestimation of portions and resulting in food waste. Attempts to contact the other teams appear to be unsuccessful. An employee told that they do not get a response when attempting to provide feedback on missing meals via mail.

*“We emailed them [Kitchen staff] every day last week about disposables missing and well… we got no response.” (Respondent 2, Wait Staff Loc. B)*.

“*When you think about it: these decisions are about food, it’s about sustainability, and the future. Why are kitchen staff not actively involved decisions made by facility management?” (Respondent 10, Kitchen Staff)*.

Employees indicated that they have limited understanding of the work duties and responsibilities of their colleagues from other teams. This lack of transparency in terms of work activities contributes to misunderstandings and misconceptions among staff members.

*“Yes, they [kitchen staff] uses quite a lot of cans and readymade mixes products. Not everything, but it happens often.” (Respondent 7, Wait Staff Loc. B)*.

*“No, no, no, everything we make is freshly prepared.” (Respondent 1, Kitchen Staff)*.

Some staff members reported not having visited the other location that is across the street: *“I have never been to location A at all. So, I really have no idea”.* These misunderstandings and lack of knowledge about colleagues’ work were identified by the staff members as key factors in the low levels of accountability, cooperation and trust observed between different teams. Staff members who share a physical building had a more positive perception of each other, indicating that increased interaction and collaboration may be beneficial in fostering better communication and understanding among staff members.

### Theme 2: understanding and knowledge of the concepts healthy and sustainable

The perception of a healthy diet for older adults among staff members varied. The most commonly cited aspect of a healthy diet was a balanced and varied menu. Instead of providing a direct response to the asked question of what a healthy diet is, staff members often emphasized the importance of creating a pleasant social experience as opposed to focusing on the nutritional content of the provided food. Only one staff member spontaneously addressed the specific nutritional needs of older adults, such as recommended protein consumption and intake of fruits and vegetables, and mentioning the importance of reducing the salt intake. Some staff members even prided themselves that prevalence of malnutrition was not present among the residents of their location, however, this was not accurate.

*I: “Do people with malnutrition live here?” R: “No [in amazement]. That does not exist here. Definitely not here in this house.” (Respondent 4, Wait Staff Loc. A)*.

*Healthy is simply a balanced meal. Just a good piece of meat with vegetables and potatoes or rice. (Respondent 14, Kitchen Staff)*

In contrary, the perception of diet sustainability among staff members was limited and fragmented. Staff members had varying and inconsistent understandings of the concept of sustainability, with some only focusing on specific aspects such as packaging and disposable use, while others discussed sustainability in relation to the type of consumption, such as organic or less meat, and reduction of food waste. Some staff members had limited understanding of sustainability in general, and were unable to provide a clear understanding of how it applies to food. After being prompted on the topic of sustainability, all teams voiced a light awareness towards the need to combat food waste and reduce the use of disposable items. In addition, when asked for a healthier menu the suggestions were mostly “*more vegetables and fresh*”.

*“Sustainable food, what is sustainable food? I must think for a moment… I mean, really, I don’t know what to say.” (Respondent 7, Wait Staff Loc. B)*.

*“Desserts go in plastic containers; meals go in those black plastic disposable trays. Well, we have quite a bit of food waste.” (Respondent 14, Kitchen Staff)*.

Despite the lack of understanding the abstract concepts of healthy and sustainable, these staff members were expected to play a key role in transitioning towards a more healthy and sustainable menu. While facility management staff had a clear understanding of the desired changes to be made towards a healthier and more sustainable food system, other staff members did not have the same level of understanding. However, kitchen and waiting staff did not perceive a lack of knowledge on their part, but rather a lack of clear and tangible guidelines from facility management.

Staff members expressed confidence in their ability to play a role in the transition towards a healthier and more sustainable menu. Several minor initiatives were mentioned by staff members, particularly by wait staff of both locations. Despite this confidence in themselves, many staff members also reported low trust in some of their colleagues, describing them as resistant to change and stuck in traditional practices. Additionally, wait staff tended to have a negative perception of the abilities of kitchen staff and the food they served, indicating low trust in the practices of their colleagues.

*“I removed the molten butter sauce from the menu; I thought it was just fat and unhealthy for those people.” (Respondent 3, Wait Staff Loc. A)*.

*“It is all so rusted shut, new ideas had to be brought in. Some cooks have been working here for 100 years, but we also have a few guys who really enjoy trying something different.” (Respondent 11, Kitchen Staff)*.

### Theme 3: a pampering service mind-set

All teams reported a shared goal of providing the highest quality of life for the residents, whom they viewed with sympathy due to their old age and frailty. Kitchen staff emphasized their focus on preparing delicious and varied meals for the residents, while wait staff sought to pamper the residents by offering extra snacks, side dishes, and beverages, and placing emphasis on food presentation. The clear motivation and objective of the staff members could serve as a facilitator in the transition towards a healthier and more sustainable food environment. However, this common attitude of pampering the residents could also have negative effects, as one facility manager noted.

*“Because we are not a hotel. No, we are not a hotel, despite we sometimes behave like one.” (Respondent 5, Facility management)*.

There was a clear pattern in the responses to the question of what staff members believed to be healthy food for older adults. Interviewees considered serving “*healthy foods like extra vegetables*” cruel or unnecessary for residents in the later stages of life. Staff members often stated that they provide residents their own freedom of choice and that if residents want to eat healthy, they can ask for additional portions of vegetables. This approach reflected the desire of wait staff to avoid coddling the residents, who have already lost a significant degree of autonomy in the residential care setting they are in. Staff members emphasized the importance of variation in the food options, as the freedom of choice for residents is limited by the supply-driven structure of the food service. However, this attitude of not wanting to interfere in the residents’ food choices also absolved staff members of responsibility for promoting healthy and sustainable food options. Staff members, in particular wait staff, were observed to actively and continuously offer unhealthy snacks such as cookies, wine, and coffee to the residents.

*“For me, it is important to have that salad available. But most people chose applesauce anyway because they find it of course much tastier. But hey, we still give them the choice.” (Respondent 3, Wait Staff Loc. A)*.

*“We’re not going to deprive those people of anything, maybe they’ve only got four weeks to go. Enjoy!” (Respondent 1, Kitchen Staff)*.

### Theme 4: transition is important but hard to realize

The presence of entrenched daily practices and existing routines may lead to staff members becoming comfortable with traditional practices and resistant to change. For example, kitchen staff served custard 7 days a week because it was considered “*nice and easy”* and “*it’s always been like that’.* Some staff members were concerned about negative reactions from residents if they deviated from these traditional practices, as residents were perceived as “*so spoiled and used to it*.“ The staff members who were resistant to change cited negative past experiences and did not see the benefit of changing established practices. Conversely, other staff members were open to breaking with tradition and exploring new options. Wait staff anticipated positive reactions from residents when trying new types of food, such as couscous.

*“So, in addition to variation in the preparation method, we can also try different products. Not always just potatoes but come on! Try something new like couscous or bulgur. That is possible. Maybe they [the residents] really like it, but if we do not serve it to them, we do not know. And if they don’t like it, we just don’t do it again.” (Respondent 8, Kitchen Staff)*.

Facility management and kitchen staff emphasized that the success of the transition towards a healthier and more sustainable food system also depends on cooperation from suppliers. They viewed reducing food waste and the use of disposable items as being outside of their sphere of influence and therefore not their responsibility. They indicated that if suppliers are unable to decrease the amount of disposable items, it is beyond their control. On the other hand, wait staff members viewed the responsibility as being dependent on internal factors such as the participation of other teams within the food service chain. They stated that they receive food from the kitchen staff and do not have any control over what is delivered, which leads them to perceive that the responsibility for sustainability and healthiness of food falls on the kitchen staff.

*“We need a supplier where we return our used packing material. The dairy comes in buckets, we have about six plastic buckets left every day. They must be taken back. That should be possible, right?” (Respondent 1. Kitchen Staff)*.

*“No, because that’s how we get our food delivered, and we have to deal with it.” (Respondent 13, Wait Staff Loc. B)*.

Finally, the kitchen staff perceived economic constraints, limited staff resources, and time as limitations to making changes towards a healthier and more sustainable menu. These perceived barriers appear to be in contrast with management policies that state that they support new ideas with resources such as time and money. Additionally, kitchen staff identified practical barriers such as the capacity and equipment limitations of the kitchen as impeding their ability to successfully implement changes.

## Discussion

This qualitative study aimed to investigate the barriers and facilitators that are perceived by the nursing home food service staff members to establish a transition towards a healthier and sustainable food environment. Results from this study show that from the perspective of wait staff, kitchen staff, and facility management, it is necessary to consider: (i) the nature and effectiveness of communication flows, including feedback mechanisms, (ii) the actual understanding, knowledge and abilities of staff members, (iii) the presence of a cohesive organizational attitude and commitment, and (iv) sufficient willingness to change and resources to support the transition.

Consistent with previous research in health care settings, staff members reported that time, money, and personal resources were major barriers to implementing changes [20, 36, 37]. Additionally, this study revealed that a lack of transparency and accountability within the food supply chain, for example the food waste, was a barrier. Each team works on its own part of the food supply chain and interaction between the teams was minimal. The teams felt successful in achieving their own goals, when they do not experience any direct disadvantages from not committing to the broader sustainability goals such as reducing food waste or implementing more healthy options in the menu. Managers can support the transitions process by providing clear, transparent communication procedures among the work teams [37].

A new insight emerging from this study includes the finding that staff are motivated by the satisfaction of the residents they care for, as this emerged as a common theme throughout the interviews. Specifically, if those residents are also satisfied with a shift towards healthier and more sustainable food options. Another new insight of this study is the lack of understanding of the concepts healthy and sustainable among staff members. Despite the high self-efficacy and perceived knowledge among staff, responses to questions about healthy and sustainable food, in general and for older adults, were inconsistent, limited and sometimes incorrect [24, 38]. Not grasping abstract subjects like nutritional health and sustainable food could be attributed to the lower levels on education of the kitchen and wait staff. This is not a skill set they normally have to master within their job.

The pampering mindset among staff members was expected as previous research showed that the meal event in nursing homes does not only serve the purpose of providing nutrients but it is strongly tied to cultural identities and relationships [39]. The food service staff members indicated the importance of maintaining residents’ freedom of choice because their autonomy is already limited due to the nature of a ‘closed setting’. These findings are in line with previous research, whereby it was concluded that nursing home staff play a key role in supporting resident autonomy and thereby promoting their quality of life [40].

Results revealed that kitchen staff were the most resistant to change, which was anticipated as menu changes would have a greater impact on their daily routines compared to wait staff. Emphasizing the personal benefits, such as the goals of kitchen staff to deliver a good product and wait staff a pleased resident, may increase commitment to the transition. Studies have shown the importance of staff members being convinced about the intervention’s value for a successful implementation [41, 42] and in many instances of transition, those who stand to gain from the change (namely, management and patient health) often differ from the group that experiences the most significant impact on their workflow (such as kitchen and serving staff) [43]. However, previous research has shown that strong leadership might be a major precondition for starting a transition, for receiving necessary resources, for ensuring the liability and seriousness of the transition, as well as for a durable implementation [44].

The MOA framework is used as a foundation for the preparation of the interview guide and analysing the data [25]. The MOA framework is well established as a theoretical basis for the explanation of work performance [45]. The findings support the MOA framework. The food service staff needs the ability to understand what the transition towards a healthy and sustainable food environment entails. Food service staff who perceive to be not equipped with sufficient resources have difficulty participating in the transition. Another important factor is that the food service staff must identify the opportunity; staff members not sensing the usefulness of the transition do not have an urge to cooperate in the transition, and thus not find it an opportunity. Food service staff must see a benefit for themselves, intrinsic motivation, to make the transition succeed. However, Aramyan and colleagues conclude that motivated, inspiring frontrunners are of key importance in the initial phase of a transition process [46].

### Strengths and limitations

The strengths of this study include the inclusion of multiple perspectives from wait staff, kitchen staff, and facility management, as well as a focus on staff members rather than residents. Prior research on food service staff in nursing homes has often underrepresented the perspectives of kitchen staff and focused primarily on nursing staff and residents [42, 47–49]. This study also provides a comprehensive understanding of the food supply chain in nursing homes by mapping the practices of all food service staff. Furthermore, the study-design with semi-structured interviews allowed for in-depth understanding through follow-up questions. However, some methodological considerations should be noted. Potential limitations include the possibility of socially desirable answers during interviews, although the interviews were held with no one else present. Besides, not all approached staff members wanted to participate in the interviews, therefore this study may not fully capture the range of views and perceptions among all staff members.

### Implications for future research

The current study has highlighted the perceptions of staff members regarding the barriers and facilitators to establishing a transitions towards a healthier and sustainable food environment in nursing homes. The findings suggest that future studies should focus on ways to improve the joined accountability for a transition, as well as transparency in the communication flows and how to preserve residents’ freedom of choice. Additionally, the findings suggest that the staff’s mindset of ‘*pampering residents’* can be used as a facilitator if utilized correctly. Nudging, psychological pushes in the right direction, may be used as a tool for this [50, 51]. Previous research on nudging in nursing homes showed a positive effect on food waste by changing the colour of plates on which the food was served [52]. The effect of social nudges on reducing food waste in nursing homes remains, to our knowledge, unexplored.

## Conclusion

In conclusion, this study aimed to identify the barriers and facilitators to the transition towards a healthy and sustainable food environment in nursing homes as perceived by staff members. Through thematic analysis of semi-structured interviews, we found that transparent communication, accountability in the food supply chain, staff members’ perceived ability, a shared common goal among all staff members, and adequate resources were facilitators to the transition. Additionally, barriers identified included lack of understanding and knowledge of the concepts of healthy and sustainable food, a pampering mindset among staff members, and top-down decision making. These findings provide valuable insights for closed settings seeking to transition towards a healthier and sustainable food environment.

### Electronic supplementary material

Below is the link to the electronic supplementary material.

Supplementary Material 1

## Data Availability

The datasets used and/or analysed during the current study available from the corresponding author on reasonable request.
