# Supplementary material for: The perceptions of food service staff in a nursing home on an upcoming transition towards a healthy and sustainable food environment: a qualitative study
Source: BMC Geriatr. 2023 Nov 28;23:784. doi: 10.1186/s12877-023-04493-x (PMC10685581; doi:10.1186/s12877-023-04493-x)
Supplement: Supplementary file 1 — Supplementary Material 1 [file 12877_2023_4493_MOESM1_ESM.docx]

Supplementary file: Topic guide interviews

The topic guide used during the semi-structured interviews is presented below.

| INTRODUCTION | |
| --- | --- |
| *Begin the conversation with the provided information. Maintain the indicated order.* | |
| Introduction | Introductions  We are pleased that you are willing to participate in this interview and research. I believe we've previously discussed conducting this interview with you. |
| Research Purpose | With this interview, you are taking part in a research study. We will be visiting Pennemes and Mennistenerf to interview all food service staff.  Through this research, we are exploring your experiences regarding the food offerings and identifying opportunities for further improvement towards a more healthy and sustainable menu.  Your story is important for this study; it contributes to insights in the staffs perceptions. During this interview, we will primarily be listening to you. |
| Anonymity and Confidentiality | This interview is anonymous, meaning that everything you share with me will not be disclosed. Therefore, what you say to me is confidential, and I will not share it with anyone else. |
| Early Termination | If you decide during the interview that you no longer wish to continue, you are free to indicate so at any time. We will then conclude the interview. |
| Recording | As you can see, I have brought recording equipment to capture this conversation.  According to the law, we are required to ask for your consent before using recording equipment. Therefore, I will ask you shortly, once the recorder is running, if you give permission for this conversation to be recorded.  **Are you comfortable with this conversation being recorded on tape?** |
| Assigning Tasks | I will conduct the interview with you. |
|  | **Do you have any questions before we proceed?** |
| Information | - Name: - Date: - Time: - Location: |

| VARIABLES, TOPICS, MAIN QUESTIONS, AND SUBQUESTIONS | |
| --- | --- |
| *All variables, topics, and main questions must be covered.* | |
| GENERAL  Topics:   - Name - Gender - Job (Duties) - Previous Tasks | **Main Questions:**   - **Could you tell us a bit about yourself?** - **What kind of tasks do you perform here?**   **Subquestions:**   - What tasks do you have? What responsibilities are associated with them? - Have these tasks changed over time? (→ adjustments in food vision implemented) - What aspect of working at this facility appeals to you the most? |
| LOCATION  Topics:   - Pride - Importance | **Main questions:**   - What is the facility proud of? - What aspects do you consider important? |
| CURRENT FOOD OFFERING  Topics:   - Current status - Satisfaction - What's missing - What's going well - Resident feedback - Variety | **Main Questions:**   - What do you think of the menus offered in the midday restaurant? - If you could change something about the food offerings for breakfast, lunch, and dinner, what would it be?   **Subquestions:**   - What is going well at the facility? - Which aspects would you like to see more frequently? What receives positive resident feedback for breakfast/lunch/dinner? - Which aspects are lacking? What receives negative resident feedback for breakfast/lunch/dinner? - Do you find the offerings to be varied? |
| HEALTH    Topics:   - Healthy eating - Malnutrition - Nutrient deficiencies - Overweight - Quality of life - Food groups - Vegetables - Fruit - Role in health - Resident opinions - Areas for improvement - Possibilities | **Main Questions:**  What do you consider "healthy eating" for the residents?  **Subquestions:**   - Do you think older adults need additional elements to eat healthily?   - Malnutrition   - Nutrient deficiencies   - Overweight   - Quality of life   - Food groups like vegetables and fruit - Do you believe the food served here is healthy?   - Resident opinions - What role does your position play in this? - What could be made even healthier? - Residents mentioned that they sometimes wanted to eat fruit but couldn't peel it themselves and felt hesitant to ask staff for help.   - Would it be possible to provide peeled fruit or offer an alternative?   - What would you need to implement such changes? |
| SUSTAINABILITY    Topics Kitchen staff:   - Sustainability - Food waste - Portion sizes - Freshness - Seasonal - Local   Topics wait staff:   - Sustainability - Food waste - Influencing choices - Portion sizes - Seasonal - Local | **Main Questions:**  Besides health, the food vision also aims for a more sustainable menu. Do you have an idea of what a sustainable menu might entail?  **Subquestions:**   - Could you elaborate on your concept of a sustainable menu? - If yes: Do you think a sustainable menu is feasible or suitable for the residents? - If not: sustainability focuses on reducing food waste, using local products, and emphasizing seasonal ingredients. Do you find this feasible?   **KITCHEN STAFF**  **Main Questions:**   - Do you have any ideas for reducing food waste? - Are you actively working on this? How do you prevent excess food?   - How?   **Subquestions**:   - How do you feel about the portion sizes offered to residents? - Do you believe they align with residents' preferences? - Have portion sizes been adjusted recently?   - If yes, why? - Are pre-packaged ingredients used in cooking, such as instant mashed potatoes or soup bases?   - If yes, which ones? - Would it be feasible to prepare these items from scratch?   - Any suggestions for improvements? - Is the season considered when choosing vegetables to serve? - Would it be possible to incorporate more seasonal ingredients? - Are locally sourced products used in cooking? - Would you like to work more frequently with locally made/grown products?   - If yes, any suggestions?   **WAIT STAFF**  **Main an subquestions:**   - Do you have any ideas for reducing food waste? - Are you actively working on this?   - If yes: How do you prevent excess food? - Do you guide residents toward specific menu choices?   - If yes: How? Why? - How do you feel about the portion sizes offered to residents for breakfast, lunch, and the bread meal?   - Do you receive resident feedback about portion sizes?   - Any suggestions for portion size adjustments? - Have you noticed changes in portion sizes over the past year and a half?   - What are your thoughts?   - Have there been any reactions to these changes? - How would you feel about using more local and seasonal products?   - Do you think this is feasible? |
| TRANSITION    Topics:   - Motivation - Progress - Challenges - Facilitators | **Main Questions:**  I've asked several questions about serving local, fresh, and seasonal food, as well as your perspective on healthy eating for the residents.   - Have you thought about these topics before this interview?   - Sustainability   - Health   **Subquestions:**   - If yes:   - What have you done with these ideas?   - Have you taken any actions?   - Why or why not? - If not:   - Would you like to learn more about these topics?   - Do you find them important to address?   - Why or why not? |

| CLOSURE | |
| --- | --- |
| Interview Conclusion | - Would you like to add anything else to this conversation? - Are there any important points that haven't been covered in the interview but you feel are worth mentioning? - What are your thoughts on the interview? |
| Member checking | The interview will be transcribed. If you would like, you can receive the transcribed transcript and provide comments.    A presentation of the findings will be given in August. |
| Thank You | I'd like to express my gratitude for your time and participation in the interview. I hope you feel that you've been able to share your story and that your narrative has been heard. |
| Questions | If you have any further questions, feel free to reach out to me, Femke Hoefnagels. |
